# Platelet – Leukocyte Interactions: Multiple Links Between Inflammation, Blood Coagulation and Vascular Risk

**DOI:** 10.4084/MJHID.2010.023

**Published:** 2010-08-13

**Authors:** Chiara Cerletti, Giovanni de Gaetano, Roberto Lorenzet

**Affiliations:** 1 Laboratory of Cell Biology and Pharmacology of Thrombosis and; 2 Laboratory of Thrombosis and Cancer Research, Research Laboratories, “John Paul II” Center for High Technology Research and Education in Biomedical Sciences, Catholic University, Campobasso, Italy

## Abstract

The aim of this review is to summarize the contribution of platelets and leukocytes and their interactions in inflammation and blood coagulation and its possible relevance in the pathogenesis of thrombosis. There is some evidence of an association between infection/inflammation and thrombosis. This is likely a bidirectional relationship. The presence of a thrombus may serve as a nidus of infection. Vascular injury indeed promotes platelet and leukocyte activation and thrombus formation and the thrombus and its components facilitate adherence of bacteria to the vessel wall. Alternatively, an infection and the associated inflammation can trigger platelet and leukocyte activation and thrombus formation. In either case platelets and leukocytes co-localize and interact in the area of vascular injury, at sites of inflammation and/or at sites of thrombosis. Following vascular injury, the subendothelial tissue, a thrombogenic surface, becomes available for interaction with these blood cells. Tissue factor, found not only in media and adventitia of the vascular wall, but also on activated platelets and leukocytes, triggers blood coagulation. Vascular-blood cell interactions, mediated by the release of preformed components of the endothelium, is modulated by both cell adhesion and production of soluble stimulatory or inhibitory molecules that alter cell function: adhesion molecules regulate cell-cell contact and facilitate the modulation of biochemical pathways relevant to inflammatory and/or thrombotic processes.

## Introduction:

At sites of inflammation, infection or vascular injury, local pro-inflammatory or pathogen-derived stimuli render the luminal vascular endothelial surface attractive for platelets and leukocytes. This cell response consists of a well-defined and regulated multi-step cascade involving consecutive steps of adhesive interactions between blood cells and the endothelium. Platelets arrive early at sites of inflammation contributing to both coagulation and to the immune response, in part by facilitating leukocyte-endothelial interactions. Platelets have thereby been implicated in several inflammatory disorders. During the initial contact with the activated endothelium and platelets, leukocytes roll along the endothelium via a loose bond which is mediated by selectins. This preliminary step results in the activation of leukocyte integrins and the firm leukocyte arrest on the endothelium. After their firm adhesion, leukocytes may pass the endothelial barrier. In addition to the injured vessel wall, circulating blood cells, reaching the sites of inflammation, contribute to blood coagulation by facilitating availability of vascular or blood-borne tissue factor.

It is currently accepted that thrombosis – the most common cause of ischemic cardiovascular disease, such as myocardial infarction and stroke –is the late complication of atherosclerosis, a progressive inflammatory disease characterized by lipid infiltration in the wall of large arteries (atherosclerotic plaques). Platelet and leukocyte recruitment on endothelial cells constitutes an early mechanism of vascular inflammatory damage and consequent plaque formation and vessel occlusion.^1^ Plaque rupture and thrombosis are the major causes of myocardial infarction and sudden cardiac death; the mechanisms of lesion vulnerability, based on the cellular and matrix contribution to tissue proteolysis, are responsible for thinning and rupture of the fibrous cap.^2^ During inflammation, signalling cascades result in activation of endothelial cells, platelets and leukocytes. The complex interaction between these vascular cells is influenced by both cell adhesion and production of soluble stimulatory or inhibitory molecules that alter cell function: an array of cell-cell adhesion molecules regulates this close relationship which favours the modulation of the biochemical pathways of these cells, while the interaction among soluble molecules triggers blood coagulation, a fibrin mesh is formed, and the resulting clot limits or stops the loss of blood. The net effect of this cellular cross-talk on inflammation depends on the balance between inputs and can lead to resolution and repair or perpetuation of inflammation, atherosclerosis and thrombosis.^3^–^6^ In this review, we will focus on the mechanisms of vascular and blood cell interactions, that are relevant for thrombosis and inflammation and their physio-pathological relevance, based on both experimental and epidemiological data.

## Cell-Cell Interactions In Thrombosis

### Adhesion of platelets and leukocytes to endothelial cells:

The first description of rolling of blood cells along the endothelial surface of venules was reported more than 160 years ago when leukocytes were shown to adhere to blood vessel walls, an interaction that increased in inflammation.^7^ Giulio Bizzozero in 1882 first described platelets as a new blood corpuscle, playing a relevant role in thrombosis and haemostasis, and observed that “Every time when a vascular wall is damaged… arrest of white blood corpuscles represents a secondary phenomenon and may, perhaps, be caused by increased stickiness of blood platelets whereby the latter react with white blood corpuscles which have been brought in contact with the former by blood circulation”.^8^

Many decades later, the modern version of intravital microscopy allowed the first quantitative observations of leukocyte rolling in the cheek pouch of hamsters and in mouse mesentery.^9^

Platelet adhesion to and leukocyte rolling on endothelium are the initial stage of a multistep process leading to extravasation of white blood cells to sites of inflammation or infection, to platelet-leukocyte interaction and aggregation on a thrombogenic surface and finally to vascular occlusion. Platelets may interact with endothelium even in the absence of any apparent morphological damage. They may stick indeed to an apparently intact endothelium inflamed by different stimuli, such as infection, mechanic alteration or ischemia and reperfusion or to endothelium located at lesion-prone sites, such as the carotid artery bifurcation. The recruitment of platelets and leukocytes at sites of vascular injury is a very rapid response and is mediated by the release of preformed components of the endothelium, including Weibel-Palade bodies and their major constituents, the von Willebrand factor’s largest multimers and P-selectin; these are the most active promoters of platelet and leukocyte adhesion. P-selectin mediates both leukocyte and platelet adhesion and during secretion fuses with the endothelial plasma membrane. The process of leukocyte rolling is initiated by P-selectin secretion and is concluded by leukocyte adhesion and transmigration to inflamed tissue.^3^–^5^,^10^,^11^

**Figure 1.**
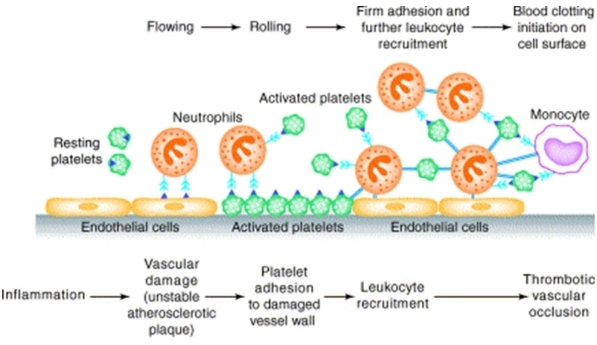
Schematic sequence of interactions between endothelial cells, platelets and leukocytes. Reprinted from de Gaetano et al,^11^ with permission of the Publisher.

The schematic sequence of interactions between endothelial cells, platelets and leukocytes flowing in blood, that lead to vascular damage and thrombus formation, is depicted in Figure 1.

Platelets activated at the site of vascular damage play a pivotal role in polymorphonuclear leukocyte accumulation in a growing thrombus and may replace endothelial cells in the recruitment and migration of leukocytes through damaged vessel wall. Platelets, on the other hand, may participate in inflammatory responses, for instance in the autoimmune disease rheumatoid arthritis by providing proinflammatory microparticles, formed upon collagen-induced platelet activation,^12^ or in cutaneous arthus reaction by controlling cutaneous inflammation via P-selectin-PSGL-1.^13^ Platelet α-granules contain and release P-selectin on their surface upon activation; since the density of P-selectin on activated platelets is much higher than that on endothelium, leukocytes are easily recruited onto the activated platelets either adherent to the endothelium or in suspension. They roll on platelets and after activation transmigrate, or accumulate in a platelet thrombus. It should be mentioned that platelet-monocyte complexes show increased transmigration compared to platelet-free monocytes.^14^,^15^ Leukocytes can contribute in turn to further platelet activation and to increased fibrin deposition.^11^,^16^,^17^

Early studies revealed that platelets, in *in vitro* co-culture experiments, provide a cholesterol-donating activity to peripheral blood monocytes-derived macrophages, enhancing both the rate of cholesteryl ester formation and accumulation.^18^ Platelet factor 4 (PF4), which is stored in platelets and released upon activation, induces monocytes to macrophages differentiation and favours foam cell development through increased oxLDL uptake by macrophages.^19^ In addition, as shown by Daub et al,^20^ platelets can induce the transformation of CD34^+^ progenitor cells into mature foam cells. In this respect, the close association between platelets and macrophages in the early fatty streak lesion might favour the formation of foam cells and, consequently, the progression of the lesion. These events may on one hand, favour the maintenance of vascular and tissue integrity, on the other, play a pathogenetic role in inflammatory and thrombotic disease. A number of data provide indeed biological plausibility to the epidemiological evidence of a significant association between leukocyte count and the incidence of coronary heart disease.^21^–^23^

### Sequence of molecular events following P-selectin expression:

P-selectin is a type-1 membrane glycoprotein with a C-type lectin domain, stored in the Weibel-Palade bodies of endothelial cells and in the α-granules of platelets.^24^ The secretion of Weibel-Palade bodies initiates a multistep process of leukocyte recruitment. In the systemic microvasculature, leukocytes are captured and begin to roll on P-selectin, expressed in acute rapid injury, and on E-selectin expressed in inflammatory conditions; in specialized endothelium, such as lymph nodes, initial interaction can be mediated by other molecules, such as L-selectin. Selectins recognize a specialized fucosylated sialoglycoconjugate, such as the tetrasaccharide sialyl Lewis X that decorates selected surface glycoproteins. The most studied ligand is P-selectin glycoprotein ligand-1 (PSGL-1), enriched at the very tip of leukocyte microvilli, which plays a major role in the initial capture of leukocytes to the endothelium. PSGL-1 and the recently described E-selectin ligand contribute to leukocyte capture on endothelium, while CD44, found on leukocytes, controls rolling velocity and signal transduction. Slow rolling allows leukocytes to interact with chemokines produced by inflamed tissues, further contributing to leukocyte activation. Recruitment specificity may be provided or enhanced to certain tissues by unique combination of adhesion receptors and chemokines. As inflammation progresses, leukocyte rolling velocity decreases, allowing the integration of activation signals from selectin ligands and heterotrimeric G-protein-coupled receptors. Downstream signal effectors include phosphatidylinositol 3-kinase (PI3K), phospholipase C, tyrosine kinases from different families and small GTPases. These signals lead to the polarization of slowly rolling leukocytes and clustering of L-selectin and PSGL-1 to a major pole that allows further leukocyte recruitment through secondary leukocyte-leukocyte interactions. P-selectin- and E-selectin-induced signals may also collaborate with chemokine receptor signalling to activate integrins.^5^

### P-selectin- β2-integrin cross-talk:

Integrins are heterodimers formed by an α and β chain, that are normally found in a closed low-affinity conformation on leukocytes. Different conformations, with matched affinity states, exist and leukocyte activation results in increased integrin affinity and avidity (strength of adhesiveness), leading to firm adhesion on endothelial intercellular adhesion molecule-1 (ICAM-1) and vascular cell adhesion molecule-1 (VCAM-1). Adherent leukocytes continue to migrate along the vascular wall, until they transmigrate either through the cell junctions among different adhesive molecules, or through the endothelial cell itself.

Leukocytes are recruited on activated platelets with molecular mechanisms similar to those just described, occurring at the site of vascular inflammation.^11^

A variety of agonists can activate platelets and induce α-granule release and P-selectin expression on platelet surface: products of vascular damage (multimeric von Willebrand factor), subendothelial collagen, traces of thrombin, the end product of the coagulation cascade, ADP, released by red cells or by activated platelets, platelet activating factor (PAF) or active products of the arachidonic acid metabolism, such as thromboxane A2, proteases released by activated polymorphonuclear leukocytes, such as cathepsin G, chemokines or other inflammatory mediators. Activated platelets within the circulation bind to leukocytes, forming platelet-leukocyte conjugates, through P-selectin interaction with PSGL-1, by a rapidly reversible tethering. PSGL-1 engagement induces in leukocytes a signal that facilitates β2-integrin adhesiveness, through involvement of activation of tyrosine kinases, mainly of the Src family, and allows the remodelling of cytoskeleton-β2-integrin linkages and clustering, that finally strengthen cell-cell adhesion. At variance with the adhesive molecules involved in rolling, which do not require activation, β2-integrins require functional up-regulation to become competent to bind their counter-receptor. The leukocyte β2-integrin, CD11b–CD18 (or Mac-1), by binding to platelet counter-receptors, such as fibrinogen, ICAM-2 and GpIbα, can stabilize the interaction with platelets, but can also interact with ligands on other cells, such as endothelial cells or different classes of leukocytes, allowing multicellular interactions.^10^

### Other adhesive systems:

In the past decade other two adhesive proteins, both relevant for cell-cell interactions, have been described on platelets, namely CD40 and CD40 ligand (CD40L or CD154).^25^ The latter is translocated to the platelet surface after stimulation and may interact with its counter-receptor CD40, present on platelets and on endothelial and monocytes/macrophages, thus facilitating platelet interaction with other blood cells. The interaction CD40L-CD40, besides promoting cell-cell adhesion, induces up-regulation of several functions in monocytes, such as chemokine and cytokine secretion, expression of the pro-coagulant tissue factor (TF), up-regulation and activation of adhesive receptors and proteases and differentiation of monocytes into macrophages.^26^ Different mediators and pathways are involved in CD40 signal transduction: among the earliest detectable events after CD40 activation have to be mentioned the activation of protein tyrosine kinases, phosphoinositide-3 kinase and phospholipase Cγ2. After early biochemical changes, these signals are translated into the activation of specific transcription factors, such as NF-κB and NF-κB-like transcription factors, that drive further gene activation.^27^

Additional interactions between platelets include monocyte triggering receptor expressed on myeloid cell 1 (TREM-1) to platelet-expressed TREM-1 ligand which may promote integrin expression.^28^

More recently, SCUBE1 (signal peptide-CUB (complement C1r/C1s, Uegf, and Bmp1)-EGF (epidermal growth factor)-like domain-containing protein 1, a protein associated with platelet-endothelial interactions, was reported as a marker of platelet activation occurring during acute ischemic stroke, being virtually undetectable in healthy subjects.^29^ SCUBE-1 belongs to a novel family of proteins, is stored in the α-granules of inactive platelets, translocated to the platelet surface upon thrombin activation, released after proteolytic cleavage as small soluble fragments, and incorporated into thrombus. Since EGF-like repeats are well known to mediate adhesive interactions, the amino terminal EGF-repeats of SCUBE1 might function as a novel platelet-endothelial adhesion molecule and play a pathogenetic role in cardiovascular biology.

Table 1 lists the major adhesion molecules involved in endothelium, platelet and leukocyte interactions.

Other functionally active products (and markers) of cell-cell interactions are the transcellular metabolites of arachidonic acid. These can be either metabolites produced by cooperation between different cell types, in which monocytes provide leukotriene A4 for metabolic conversion into leukotriene C4 by platelets^30^,^31^ and calcium ionophore-stimulated neutrophil-platelet coincubations lead to the formation of lipoxin A_4_,^32^,^33^ or single cell products, such as LTB4 in leukocytes or thromboxane (Tx) B2 in platelets, produced in greater amount thanks to the presence or adhesion with a different cell.^3^,^5^,^11^

### Platelet-leukocyte interaction in blood coagulation:

Platelets and monocytes colocalize in the area of vascular injury. Indeed, such an interaction has been shown to occur in areas of vessel damage, at sites of inflammation, and at sites of thrombosis.^34^–^36^ Increasing evidence that platelets and their interaction with inflammatory cells are essentially involved in the initiation and progression of atherosclerosis have been provided in recent years.^37^ This close apposition facilitates the interactions between the biochemical pathways present in the two cell types leading to upregulation of the cell procoagulant activity. Although an early report described a platelet factor X activating activity in a purified system in which platelets were incubated with leukocytes in the presence of bacterial endotoxin lipopolysaccharide (LPS),^38^ current evidence linking platelets and monocytes with blood coagulation is related to modulation of TF procoagulant activity.

TF is a 47-kD integral membrane glycoprotein associated with phospholipids, which, upon binding to factor VII and its active form VIIa, triggers blood coagulation leading to fibrin formation.^39^ TF is constitutively expressed in extravascular cells, in a pattern consistent with the hypothesis that TF can be considered as a “hemostatic envelope”, whose duty is to minimize blood loss when the integrity of the vasculature is compromised.^40^ Although under normal conditions vascular cells do not express TF antigen or activity, these cells, exposed to mediators elaborated during inflammatory reactions, can be induced to synthesize and express TF on their membranes.^41^

Although it is widely accepted that, among the white cells, the monocyte/macrophage is the cell capable to synthesize TF, recently, in *in vitro* models, induction of TF expression in neutrophils exposed to P-selectin and fMLP^42^ or to the complement component C5a^43^ have been reported. Neutrophil TF expression is still, however, a matter of discussion: at odd with the above mentioned findings, in which the presence of mRNA coding for TF was shown,^42^ a recent report suggests that neutrophils do not express but acquire TF from monocytes.^44^ The presence of TF in platelets has also been documented.^45^ Both a surface-associated TF activity^46^ and the presence of TF in the supernatant of activated platelets^47^ were reported. In addition, the presence of TF mRNA and pre-mRNA in platelets has been demonstrated,^47^,^48^ suggesting that these cells have the necessary “repertoire” to synthesize and express TF. However, although the expression of TF on monocyte membranes, and the consequent activation of coagulation, has been shown to be involved in thrombus formation in a variety of pathological conditions,^41^,^49^ a role for neutrophils and platelet TF *in vivo* remains to be established.

Back in Seventies, Niemetz and Marcus^50^ first reported that platelets could induce TF activity in mononuclear cells and increase it when the latter cells were stimulated by LPS. Following this pioneering observation, the effect of platelets in inducing and/or enhancing monocyte TF activity in washed cell systems was later on reported by others.^51^,^52^ In a whole blood system, Amirkhosravi et al^53^ reported that stimulated monocyte TF expression was directly proportional to the platelet count and was reduced by ingestion of aspirin. Similarly, platelet up-regulation of the expression of TF procoagulant activity in mononuclear cells was significantly impaired by pretreatment with the active metabolite of the antiplatelet drug clopidogrel.^54^ Noteworthy, in addition to TF induction, platelet-monocyte aggregates increase the expression of monocyte chemotactic protein-1 and interleukin 8, proinflammatory cytokines related to the progression of atherosclerosis.^55^–^57^

The observation that TF activity correlated with the number of platelets, arachidonic acid amplified TF expression, and platelets from donors who had ingested acetylsalicylic acid were more effective, suggested a role for a platelet-derived lipoxygenase pathway metabolite. Indeed, platelet-derived 12-hydroxyeicosatetraenoic acid (12-HETE), the end product of the 12-lipoxygenase-mediated pathway of arachidonic acid, upregulated TF expression in LPS-stimulated mononuclear cells.^58^ In addition to 12-HETE, PF4, another platelet-derived metabolite, was later reported to increase TF activity in stimulated monocytes.^59^

A role for granulocytes in modulation of TF expression during platelet-monocyte interaction has also been suggested. In this respect, granulocytes were proposed to be required for optimal TF activity in LPS-stimulated monocytes coincubated with platelet: the release of the granulocyte lysosomal enzyme cathepsin G, which induces platelet activation,^60^,^61^ would increase monocyte TF activity.^62^

Contact between platelets and monocytes was required to elicit monocyte TF, suggesting the involvement of one or more adhesion molecules.^63^ Among the different possibilities, P-selectin appeared to be the optimal candidate. A role for P-selectin in the haemostatic process has been proposed in an *in vivo* system. Accumulation of leukocytes on activated platelets and fibrin formation in a Dacron graft implanted within an arterio-venous shunt in a baboon were reduced by pretreatment of the baboon with anti-P-selectin monoclonal antibodies, indicating that P-selectin *in vivo* is responsible for platelet-leukocyte binding within the thrombus, and that these leukocytes promote fibrin formation.^16^ Indeed, both highly purified P-selectin and Chinese hamster ovary cells transfected with the P-selectin cDNA, but not with E-selectin, induce monocyte TF activity and mRNA.^64^ Recently, P-selectin-dependent platelet-monocyte aggregates were reported to upregulate TF expression in a whole blood system.^57^ A role for P-selectin in mediating the stimulatory effect of platelets and granulocytes on LPS-induced TF activity in monocytes was also shown.^62^ In addition, 12-HETE, similarly to what was observed with LPS-stimulated monocytes, greatly potentiated TF expression by P-selectin-exposed monocytes.^65^

Thus, P-selectin-expressing platelets at sites of vascular injury, responsible for docking of monocytes, trigger TF synthesis and the local activation of blood coagulation will prevent further blood loss.

The discovery of what was called “blood borne TF” added a new milestone in the search for the mechanisms leading to arterial thrombosis. Giesen et al^66^ reported that the appearance of TF antigen in growing thrombi formed on pig arterial media perfused with native blood from healthy volunteers was a matter of minutes, suggesting that TF was not due to *de novo* protein synthesis but was already present in the bloodstream. Most likely this blood-borne circulating TF is associated with microparticles. It has been known for over twenty years that most of the eukaryotic cells release vescicles either during apoptosis or upon stimulation and TF-positive microparticles have been detected in fresh blood samples of healthy individuals and found increased in patients with diseases such as cancer, endotoxemia and cardiovascular disease.^67^,^68^ An intravital microscopy system for real-time imaging *in vivo* has shown a mandatory role for the P-selectin/PSGL-1 receptor pair for TF accumulation at sites of vessel injury: microparticles of leukocyte origin carrying TF and PSGL-1 would bind P-selectin exposed on the surface of activated platelets recruited at the injury site and thus contribute to the propagation of the growing thrombus.^69^,^70^

Finally, the interaction of P-selectin with PSGL-1 has been proposed to be responsible for the transfer of TF microparticles from monocytes and, possibly, granulocytes to platelets during thrombus formation.^71^

As mentioned before, platelets carry another adhesive protein, namely CD40L, which is expressed on their membrane following activation, and which binds its counter-receptor CD40 which is located to monocyte and endothelial cell membrane.^25^ It has been shown that this interaction, similarly to the P-selectin-PSGL-1 one, triggers a procoagulant response. Indeed, CD40 engagement in endothelial cells,^72^ both with fibroblasts transfected with CD40L gene and with CD40L-expressing activated platelets,^73^ in vascular smooth muscle cells,74 in melanoma cells^75^ and in monocytes^75^–^77^ results in TF expression in all the cells tested.

Since CD40 and CD40L have been detected in atheromatous plaques,^78^ it is conceivable that, in addition to plaque smooth muscle cells, activated CD40L-expressing platelets recruited at the site of endothelial damage, would tether monocytes and induce TF synthesis, thus increasing the thrombogenicity of the plaque during the inflammatory responses of atherogenesis and arterial injury.

### Soluble adhesive molecules:

After cell surface expression, some adhesive molecules, namely P-selectin, CD40L and SCUBE1 from platelets, L-selectin from leukocytes, or E-selectin, ICAM-1 and VCAM-1 from endothelium, are found in the circulation, as the result of proteolytic cleavage. The biological activity, either as activators or inhibitors of cell function, of soluble adhesive molecules detected in the circulation is not completely clarified, but their increased levels have been reported in patients at high cardiovascular risk in different studies (hypercholesterolemia, type 2 diabetes), or associated with pro-inflammatory status or cardiovascular disease.^79^–^82^

Whether the measurement of soluble adhesive molecules is merely an epiphenomenal reflection of the inflammatory and thrombotic processes, or whether it directly contributes to acute coronary events remains to be established.

### Clinical and epidemiological evidence of cell-cell interactions in cardiovascular disease:

Despite the discovery, already long ago, of the role of platelets in experimental thrombosis and of the remarkable antithrombotic effect of antiplatelet drugs in several ischemic conditions, there is still little *direct* evidence that platelets play an important role in the pathogenesis of vascular disease. This “platelet paradox”^83^ is based on the lack of clinical data showing a direct relationship between the platelet number (and/or platelet function parameters) and the number (and/or severity) of vascular events. Data showing reduced vascular risk in thrombocytopenic patients and a proportional increase of risk following transfusion of platelets are also lacking to *directly* support the role of platelets in thrombosis.

On the other hand, positive correlations between the count of leukocytes (neutrophil polymorphonuclear -PMN-, in particular) and the risk of myocardial infarction and stroke have been suggested since many years.^21^,^22^ Cardiovascular events are recognized as the most important cause of mortality and morbidity in patients with inflammatory rheumatoid arthritis.^84^ Leukocytosis has been also associated with both acute thrombosis and atherosclerosis. However, up to now no treatment reducing leukocyte function resulted effective in humans and treatment with hydroxyurea, reducing leukocyte count, has not yet been tested in patients at high ischemic risk.^23^

The hypothesis that leukocytes and platelets mutually interact and contribute to the development of thrombotic ischemic disease is taking more and more credit. Increased *ex vivo* functional responsiveness as well as *in vivo* PMN activation and platelet-leukocyte interaction in different clinical manifestations of ischemic heart disease have been reported and an active role for these cells in the progression of vascular occlusion has been suggested. Although most platelet-leukocyte aggregates have probably a short intravascular survival time, so that only a small fraction of the formed aggregates are detectable in the systemic circulation, platelet-leukocyte conjugates have been observed in peripheral blood from patients with unstable angina, stable coronary artery disease or mechanical heart valve replacement, as well as in patients with myeloproliferative disease.^3^ The formation of platelet-leukocyte conjugates following percutaneous coronary interventions was considered as a predictive index of acute re-occlusion.^3^–^5^,^23^,^24^,^85^,^86^

Recent *in vitro* work from our group^42^ indicates that neutrophil activation by platelet P-selectin results in TF expression and synthesis, a phenomenon inhibited *in vitro* and *ex vivo* by hydroxyurea in patients with myeloproliferative disease.^87^

In the framework of a large epidemiological study conducted by our group, platelet-leukocyte conjugates and their determinants were evaluated in citrated whole blood from 349 subjects (209 women, 16–92 years) randomly recruited from the general population.^88^ Platelet activation obtained by *in vitro* addition of ADP/collagen, but not leukocyte stimulation by the inflammatory peptide fMLP or by LTB4, resulted in formation of platelet conjugates with PMN or monocytes. *In vitro* stimulation with ADP/collagen significantly increased basal platelet conjugates with PMN or monocytes, platelet P-selectin and leukocyte CD11b expression. Platelet count significantly correlated with platelet-PMN, platelet-monocyte conjugates and P-selectin both at baseline and upon stimulation. In all conditions, both conjugate levels correlated with each other, when adjusted for gender, age and platelet count. Age correlated with platelet-PMN conjugate numbers in basal and stimulated conditions and with basal P-selectin. ADP/collagen stimulation resulted in higher P-selectin and conjugates values in women. Among risk factors, a significant correlation was found between cell conjugates and glucose levels. This study suggests that the presence and formation of platelet-leukocyte conjugates in citrated whole blood from a large population reflects primary platelet – but not leukocyte – activation and varies with gender, age, platelet count and blood glucose levels.

### Modulation of cell-cell interactions:

Antiplatelet agents, such as dipyridamole, the thienopyridine clopidogrel, the anti-GpIIbIIIa abciximab, or the stable prostacyclin analogue iloprost, have all been tested for their ability to modify platelet-leukocyte interactions. The control of cell-cell interactions is a new concept in pharmacology, including the modulation of cell signalling necessary for adhesive molecule expression and activation. Recently, heparins, and in particular low molecular weight heparins, have been shown to interfere with platelet-leukocyte (and tumor cell interactions too) at the level of P-selectin-PSGL-1 adhesion.^89^ The effects of old or new drugs or of diet-derived polyphenols^3^,^10^,^11^,^90^ on cellular functions and interactions relevant to inflammation and cardiovascular ischemic disease is beyond the scope of this chapter.

## Figures and Tables

**Table 1.** Major adhesion molecules in endothelium, platelet and leukocyte interactions

**Molecule**	**Origin and expression**	**Function**	**Ligand**
**Selectins**			
P-selectin	Stored in EC and platelet granules;expressed on cell surface on stimulation and released	Rolling of leukocytes on EC and platelets and of platelets on EC	PSGL-1
E-selectin	Induced by cytokines on EC	Rolling of leukocytes on EC	PSGL-1 ESL-1 CD44
L-selectin	Expressed on leukocytes	Secondary leukocyte recruitment	PSGL-1
**Immunoglobulins**			
ICAM-1	Up-regulated by cytokines on EC and leukocytes	Firm adhesion and transmigration of leukocytes	β2-integrins
ICAM-2	Constitutive on EC and platelets	Firm adhesion and transmigration of leukocytes; platelet adhesion to leukocytes	β2-integrins
VCAM-1	Up-regulated by cytokines on EC	Firm adhesion and transmigration of leukocytes	α4-integrins
PECAM-1	Constitutive on EC, platelets and leukocytes	Transmigration	PECAM-1
**Integrins**			
β2-integrins	Expressed on leukocytes; require activation	Firm adhesion to EC and platelets	ICAMs VCAM fibrinogen
β3-integrins	Expressed on platelets (αIIbβ3 or GpIIbIIIa) and on neutrophils/EC (αVβ3); require activation	Firm cell adhesion	Fibrinogen; extracellular matrix molecules
**Tumor necrosis factor family**			
CD40 CD40L	Constitutive and expressed on EC, leukocyte and platelet surface	Activates different EC, leukocyte and platelet function	CD40L CD40; αIIb β3 on platelets
